# Asymmetric interaction of point defects and heterophase interfaces in ZrN/TaN multilayered nanofilms

**DOI:** 10.1038/srep40044

**Published:** 2017-01-05

**Authors:** Yuanxia Lao, Shuanglin Hu, Yunlong Shi, Yu Deng, Fei Wang, Hao Du, Haibing Zhang, Yuan Wang

**Affiliations:** 1 Key Laboratory of Radiation Physics and Technology, Ministry of Education, Institute of Nuclear Science and Technology, Sichuan University, Chengdu 610064, P.R. China; 2 Institute of Nuclear Physics and Chemistry, China Academy of Engineering Physics, Mianyang 621900, P.R. China; 3 School of Physics, Nanjing University, Nanjing, 210093 P.R. China; 4 Division of Surface Engineering of Materials, Institute of Metal Research, Chinese Academy of Sciences, Shenyang 110016, P.R. China

## Abstract

Materials with a high density of heterophase interfaces, which are capable of absorbing and annihilating radiation-induced point defects, can exhibit a superior radiation tolerance. In this paper, we investigated the interaction behaviors of point defects and heterophase interfaces by implanting helium atoms into the ZrN/TaN multilayered nanofilms. It was found that the point defect-interface interaction on the two sides of the ZrN/TaN interface was asymmetric, likely due to the difference in the vacancy formation energies of ZrN and TaN. The helium bubbles could migrate from the ZrN layers into the TaN layers through the heterophase interfaces, resulting in a better crystallinity of the ZrN layers and a complete amorphization of the TaN layers. The findings provided some clues to the fundamental behaviors of point defects near the heterophase interfaces, which make us re-examine the design rules of advanced radiation-tolerant materials.

Materials in radiated harsh environment usually experience severe radiation damages, manifested by void swelling, amorphization, radiation hardening and embrittlement, etc.^1^,^2^,^3^. Primary radiation damages of materials usually take the form of interstitials and vacancies^4^. When interstitials and vacancies of materials induced by radiation cannot recombine and annihilate in time, they tend to aggregate separately, forming voids clusters, dislocation loops, etc., and causing hardening, embrittlement and creep of materials^4^,^5^,^6^. One approach of removing and suppressing radiation damages is to develop materials with engineered interfaces (like grain boundaries, interphase boundaries and surfaces) which can serve as sinks of point defects (interstitials and vacancies) induced by radiation^7^. For example, nanomaterials with numerous homophase interfaces (grain boundaries) can facilitate the recombination of interstitials and vacancies so that the density of point defect clusters is reduced, thus improving the radiation tolerance of materials^8^,^9^,^10^. However, the homophase interfaces tend to migrate and disappear, either during thermal annealing, or when subjected to external forces, such as radiation or severe plastic deformation, hence reducing the ability of nanomaterials to resist radiation damage^5^. On the other hand, nanostructured oxide dispersion strengthened (ODS) steels and multilayered nanofilms with heterophase interfaces (interphase boundaries), which connect two immiscible phases, not only can annihilate radiation-induced defects efficiently, but also are very stable^11^,^12^,^13^. It was reported that thermal coarsening at high temperature could be suppressed in the highly immiscible alloy systems^14^,^15^. Therefore, engineering of heterophase interfaces has been a promising approach for designing radiation tolerant materials^5^,^16^,^17^.

However, our understanding of the mechanism of the interactions between radiation-induced point defects and interfaces is rather limited^6^,^7^, especially of the exact processes by which the defect-interface interacts. Because laboratory experiments cannot directly observe these mechanisms, the atomistic simulations have been crucial in elucidating the mechanisms and processes^7^. Theoretical simulations suggested that the energy barriers for point defect migration were lower near the interfaces than those in the bulk^18^,^19^,^20^,^21^. In particular, the interstitials adsorbed or emitted by the homophase interfaces could annihilate the vacancies that come within a few nanometers of the interfaces^18^,^22^. The interaction between the point defects and the heterophase interfaces appears to be complicated. An atomistic simulation of the interfaces of Cu-Nb revealed that the absorption of point defects was not uniform on the two sides of the heterophase interfaces^21^. Cu interstitials were preferentially absorbed into the heterophase interfaces while little absorption of the interstitials on the Nb side was observed. The emission range of the interstitials from the two phases was also not uniform^21^.

Although these simulation models provide some insight into the nature of the point defects interactions with the interfaces, there is still a large gap between the atomistic simulations and implementation of the predictions in the microstructure or property in engineering processes, due to the limit of the accuracy of computer simulation in larger time- and length-scales^5^. According to some of the models^21^, the preferential absorption and emission of the point defects in different phases can induce apparent redistribution of the point defects and the changing of local chemical composition on the both sides of the heterophase interfaces. It is therefore likely to result in an undesirable transition of the microstructure of materials, which can impact the radiation tolerance and the stability of multiphase materials in radiation environments^23^. It is important to understand the responses of the microstructures of the materials to the atom behaviors in local areas at engineering-scale because the radiation tolerance of the materials might be affected. Therefore, experimental researches need to be conducted to determine the relationship between the point defect-interface interaction and the microstructure evolution.

The primary challenge of experimental research is the difficulty in the characterization of vacancies^7^,^24^. One approach in the characterization of vacancies is the implantation of Helium (He), which is insoluble in most solids, as interstitials^17^. Helium can bind strongly with vacancies in materials to form He-vacancy clusters^25^,^26^. The He bubble or void can be observed as long as the He-vacancy cluster grows above a critical size (1~2 nm)^7^. Therefore, He can be used as a tool to reveal the traces of point defects to some extent^10^,^27^.

Here, we designed ceramic multilayered nanofilms of ZrN/TaN, in which He was implanted uniformly during the films deposition, to investigate experimentally the interacting behaviors of the heterophase interfaces and the point defects. The evolution of the microstructures of the multilayered nanofilms during the process of absorption and emission of the point defects was analyzed using an X-ray diffractometer (XRD) and a transmission electron microscope (TEM). It was observed that the defect-interface interaction on the two sides of the ZrN/TaN interface was asymmetric. The helium bubbles could migrate from the ZrN layers into the TaN layers through the heterophase interfaces, resulting in crystalized ZrN layers and a complete amorphization of the TaN layers. Atomistic simulations revealed that the asymmetric defect-interface interaction stemmed from the large difference in the vacancy formation energy between ZrN and TaN. The results provided some clues to the fundamental behaviors of point defects near the heterophase interfaces, which could guide the design of more stable radiation-tolerant materials.

## Results

### Film Microstructures

**Figure 1:**
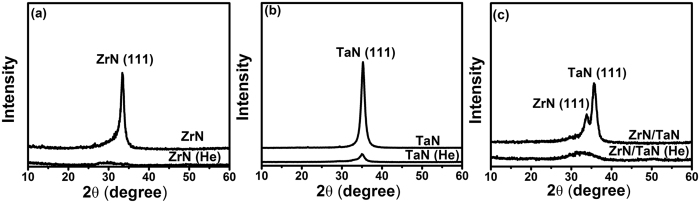
XRD profiles of different nitride films: (**a**) ZrN monofilms with and without He implantation; (**b**) TaN monofilms with and without He implantation; (**c**) ZrN/TaN multilayers with and without He implantation. The crystallinity of the all nitrides decreased with He implantation.

Figure 1 shows the XRD profiles of the as-deposited and He-implanted nitride films. It was found that the He implantation had a great impact on the crystal structures of the nitride films. As to the ZrN film without He implantation, a unique diffraction peak was found in the XRD profile (see Fig. 1(a)), implying a (111) preferred orientation in the ZrN films with a face-centered cubic (FCC) structure. However, the intensity of (111) peak decreased dramatically and could hardly be identified in the profile (Fig. 1(a)) as He atoms were implanted into the ZrN film. Similar changes of the XRD patterns were also found in both TaN (also with a FCC structure) and ZrN/TaN multilayered nanofilms (see Fig. 1(b) and (c)). These changes indicate that the amorphization of nitride films might have occurred when He was introduced during the films deposition.

**Figure 2:**
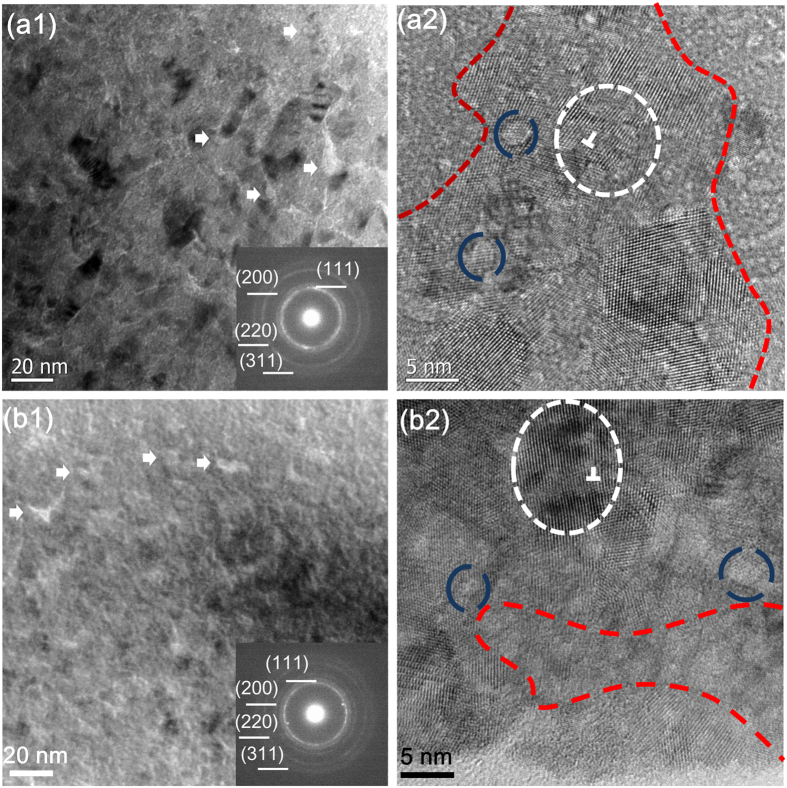
Cross-sectional TEM images of the ZrN and TaN monofilms with He implantation. A large amount of He bubbles distribute and aggregate (marked by arrows) in the ZrN (**a1**) and TaN films (**b1**), and the intact but diffused SAED patterns (inset) show the He-implanted nitride films are composed of nanocrystallines and amorphous phases. The lattice distortion and dislocations (marked by white dash circles) are commonly found in the ZrN film (**a2**) and TaN film (**b2**) with a high density of He bubbles (marked by blue circles). The amorphous areas of films (divided by red dash lines) are full of He bubbles (**a2** and **b2**).

To reveal the effects of He implantation on the microstructure of nitride films, the cross-sectional TEM images of the ZrN and TaN monofilms with He implantation are shown in Fig. 2. A large number of He bubbles were found in both ZrN and TaN monofilms, and some of the bubbles had already aggregated into larger voids (marked by white arrows in Fig. 2(a1) and (b1), respectively). The corresponding selected area electron diffraction (SAED) patterns for both nitride films with He implantation are shown at the upper right of Fig. 2(a1) and (b1). The intact but diffused diffraction rings indicate that the He-implanted nitride films were composed of nanocrystallines and amorphous phases. The HRTEM images of the ZrN and TaN monofilms with He implantation are shown in Fig. 2(a2) and (b2), respectively. It is apparent from the figures that the high density of He bubbles (marked by blue dash circles) had largely destroyed the long-rang order in the structure of the nitride films. The lattice distortion and dislocations were commonly found in both ZrN and TaN films with He implantation (marked by white dash circles in Fig. 2(a2) and (b2), respectively). Severe amorphization was found in the regions with high density of He bubbles (divided by red dash lines in Fig. 2(a2) and (b2)). It is likely that the amorphization of local area in the nitride films originated from the aggregation of He bubbles.

The defects (such as the He bubbles, voids, dislocations) and the amorphous areas induced by He implantation found in this study are similar to the radiation-induced damages found in the materials under ion or neutron radiation^9^,^28^,^29^, suggesting that the implantation of He during the film deposition could simulate the radiation-induced defects in bulk materials. With a more uniform distribution of the point defects (or clusters), the interaction between the point defects and the heterophase interfaces could be characterized in simpler terms.

### Microstructure evolution near the heterophase interfaces

**Figure 3:**
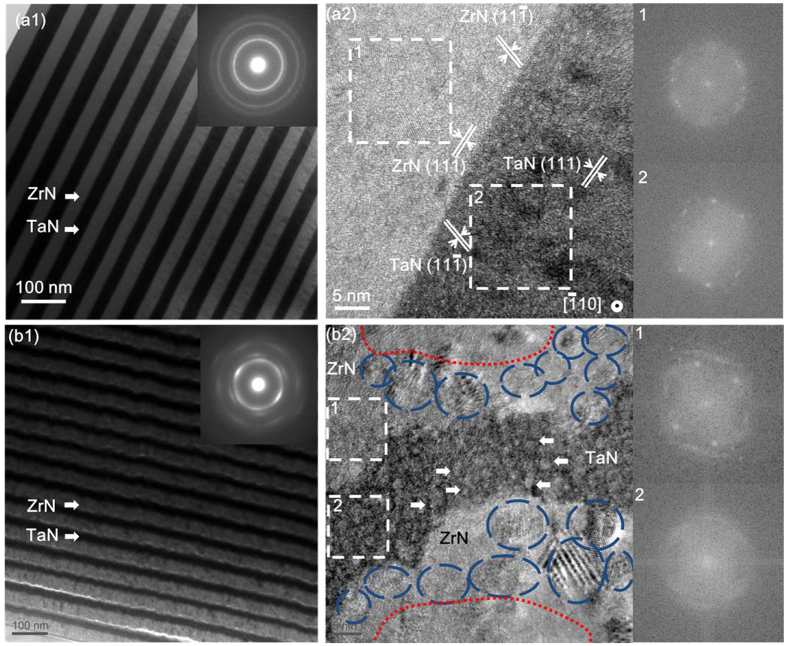
Cross-sectional morphology of ZrN-TaN multilayers with He implantation taken by TEM: (**a1**) a low-magnification image of ZrN/TaN multilayers without He implantation, and the corresponding SAED pattern; (**a2**) a HRTEM image showing the typical crystal orientation of ZrN and TaN near the heterophase interface: ZrN(111)//TaN(111)//the interface plane; The corresponding fast Fourier transform (FFT) patterns showing that both sides of the interface (marked by white rectangulars) are crystalline phases; (**b1**) a low-magnification cross-sectional image of ZrN/TaN multilayers with He implantation, and the corresponding SAED pattern; (**b2**) a HRTEM image showing the typical distribution pattern of He bubbles near the heterophase interfaces, much more He bubbles gathered on the TaN side (marked by arrows) than on the ZrN side; the FFT patterns of corresponding regions (marked by white rectangular) indicating that the TaN layer is composed of amorphous phases while the ZrN layer near the interface exhibits a better crystallinity (divided by red dash lines and marked by blue circles).

The cross-sectional TEM images of the ZrN/TaN multilayered films are shown in Fig. 3. The heterophase interfaces of the multilayered films without He implantation between the ZrN and TaN layers were rather straight (Fig. 3(a1)). This observation indicates that the ZrN and TaN layers were immiscible. The SAED pattern (Fig. 3(a1) insert) composed of some sharp and intact rings demonstrates a high degree of crystallinity in the ZrN/TaN multilayers without He implantation. The HRTEM image (see Fig. 3(a2)) shows that the grain size of both ZrN and TaN had decreased dramatically to 5–10 nm in the multilayered films. Furthermore, the typical crystal orientation of ZrN and TaN layers near the heterophase interfaces was found to be similar (Fig. 3(a2)): ZrN (111)//TaN (111)//the interface plane. This preferential growth of the texture in multilayers was in line with the XRD profiles as shown in Fig. 1(c). It can be contributed to similar crystal structure and lattice parameters of the ZrN and TaN layers. The corresponding fast Fourier transform (FFT) patterns (inset at the right of Fig. 3(a2)) confirmed that the presence of good crystallinity in the areas very close to the interface (marked by rectangles).

However, the morphology and crystal structure of the multilayered nanofilms with He implantation near the heterophase interfaces has changed drastically. The morphology of the ZrN/TaN heterophase interfaces with He implantation had become curved and irregular compared to their counterparts without He implantation. These relatively blurry heterophase interfaces (Fig. 3(b1)) signify the intermixing of ZrN and TaN layers to some extent^30^. The diffused diffraction rings in the corresponding SAED pattern (Fig. 3(b1)) indicate the formation of amorphous areas in the multilayered nanofilms with He implantation. The crystal structures of the layers on one side were also totally different from those on the other side of the heterophase interface (Fig. 3(b2)). Large amount of nanocrystallines was found on the ZrN layers near the interfaces (marked by blue circles). The corresponding FFT pattern at the upper right of Fig. 3(b2) confirmed the presence of good crystallinity of the ZrN layers near the interface because of the lacking of He bubbles aggregated on the ZrN sides. However, the uniformly distributed high density He bubbles (~2 nm in diameter) in the TaN layer (marked by arrows) likely resulted in a complete amorphization of the TaN layer (see the corresponding FFT pattern at the lower right of Fig. 3(b2)).

It is important to note that the implantation of He resulted in a similar changes in the morphology in the ZrN and TaN monofilms films (Fig. 2). However, the changes in the morphology of ZrN and TaN layers in the He-implanted multilayered nanofilms were different from that in their monofilms counterparts. The He bubbles were seldom found on the ZrN side of the ZrN/TaN interface, and its bubble density was much lower than that in the ZrN monofilm (see Figs 2(a2) and 3(b2)). In contrast, a large amount He bubbles were found on the TaN side of the ZrN/TaN interface, and the bubbles density was clearly higher than that in the TaN monofilm (see Figs 2(b2) and 3(b2)). Because the ZrN/TaN multilayers without He implantation had showed similar grain size and preferential growth in textures on both sides of the heterophase interfaces (Fig. 3(a2)), the effects of the nanocrystal grain boundaries on the density of the He bubbles in each nitride should be similar. Therefore, the abnormal distribution of He bubbles did not originate from the nanocrystalline grain boundaries. It was more likely that it came from the interaction of the heterophase interfaces and the He bubbles, which will be discussed further in the following section.

## Discussion

Since the He bubble is a He-vacancy cluster with self-interstitials at its periphery^31^, the density of the He bubbles can, to a large extent, reflect the density of vacancy-like defects in the multilayered nanofilms^32^. As reported previously, the interfaces serving as an effective sink to the point defects could assist the vacancy-interstitial recombination near the interfaces^16^,^21^. As the vacancy density near the interfaces decreases, He atoms can be absorbed and constrained by the interfaces, resulting in a high density of He bubbles on the interfaces^7^,^9^,^10^. However, in this work, much higher density of He bubbles was found in the TaN layer instead of on the interfaces of the multilayered nanofilms and in the ZrN layers (Figs 2(b2) and 3(b2)). There was also a good crystallinity in the ZrN layers because of the absence of the point defects (He bubbles). This abnormal distribution of He bubbles in the multilayered nanofilms suggests that the He bubbles might have migrated from the ZrN layers into the TaN layer through the interfaces. It might be possible that the ZrN/TaN interfaces absorbed the point defects from the ZrN layer and then emitted them into the TaN layer.

In fact, the migration of the point defects through the ZrN/TaN interfaces could stem from an asymmetric interaction between the heterophase interfaces and the point defects in different phases. The sink efficiency of the interfaces to the point defects depends strongly on the crystallographic characters of different phases, the structure of the interfaces and the mobility of the defects^33^,^34^,^35^,^36^. Therefore, the asymmetric defect-interface interaction is inevitable on the two sides of the heterophase interfaces. For example, Wei *et al*. reported that He bubbles were often found in the Cu layers instead of in the Nb layer in a radiated nanolayered Cu-Nb composite^16^. They contributed this observation to the higher mobility of vacancies in the Cu layers than that in the Nb layers. Liu *et al* investigated the defect-interface interaction in a Cu-Nb bilayer system by computer simulation^21^. Their results indicated that the vacancy formation energy was an important parameter to describe the defect-interface interaction on both sides of the heterophase interface. It was found that interstitial atoms were absorbed preferentially in the layer which had lower vacancy formation energies, resulting in more vacancies formed in the interior of the layer^21^.

**Figure 4:**
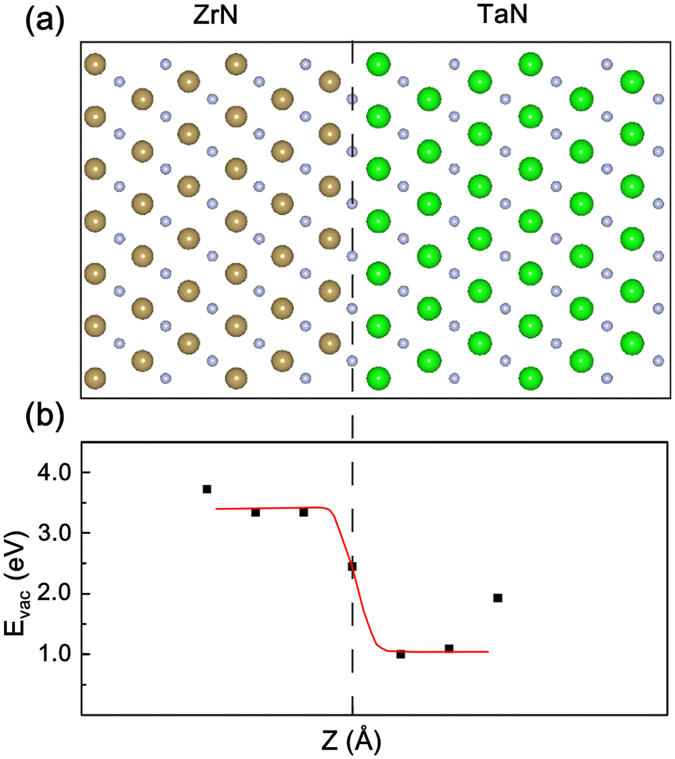
(**a**) The atomistic model of the ZrN/TaN interface before creating a nitrogen vacancy used in the calculations. The brown, green, and blue balls represent the Zr, Ta, and N. The dash line shows the position of the interface. (**b**) The calculated vacancy formation energy of nitrogen at different positions near the interface. The red line is the ideal change of the energy.

To explore the nature of the apparent distinctions of the sink efficiency on the two sides of the ZrN/TaN interface, a ZrN/TaN interface model was constructed, and a nitrogen vacancy (N_V_) site was created at several positions along the direction normal to the interface by a computer simulation as shown in Fig. 4(a). The calculated vacancy formation energies of nitrogen at different positions near the interface were also plotted in Fig. 4(b). The results showed that the formation energies of N_V_ were much lower in the TaN layer than those in the ZrN layer at positions very close to the interface. It implies that the N_V_ preferred to be created on the TaN side. It was probably resulted from a greater relaxation of atoms in TaN compared with that in ZrN when the stress was released.

It is noted that only the formation energies of N_V_ are provided here to simplify the model. In fact, N_V_ was the dominant vacancy in the NaCl-typed ZrN, while the Ta vacancy (Ta_V_) was the preferred vacancy in NaCl-typed TaN, due to its lower formation energy than N_V_^37^,^38^. The difference in the vacancy formation energies suggests that the vacancies prefer to form in the TaN layer, regardless of the sites were of N sites or of the metallic atom. The vacancies tend to migrate from the sites with high vacancy formation energy to the sites with low vacancy formation energy in order to reduce the overall energy of the whole system^38^. This explanation is consistent with the migration phenomena of point defects (He bubbles) described above.

**Figure 5:**
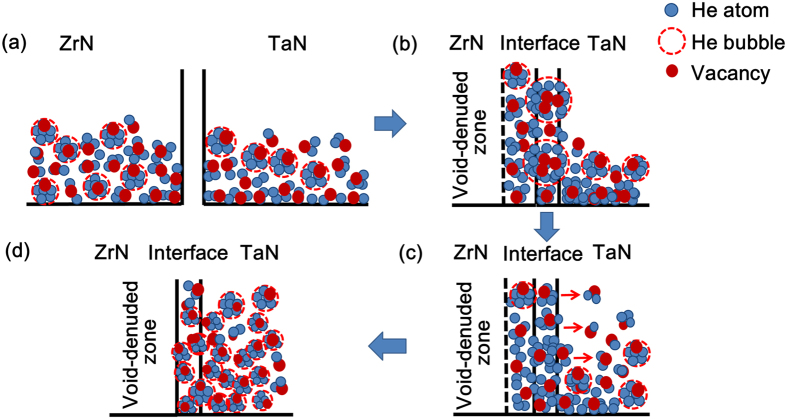
A schematic illustration of the distribution and migration of point defects in the nitride films: (**a**) distribution of point defects in the ZrN and TaN monolayers with He; (**b**) preferential absorption of vacancies and He atoms on the ZrN side of the multilayers; (**c**) preferential emission of He atoms and vacancies into the TaN layer of the multilayers; (**d**) amorphization of the TaN layer and formation of void-denuded zone (VDZ) on the ZrN side in the multilayers.

The migration of point defects (He bubbles) and the changes of crystal structure in the multilayered nanofilms are depicted in the schemes shown in Fig. 5, based on the simulation and experimental results. When the ZrN and TaN layers were deposited on the Si substrate with He implantation, a large amount of point defects including He interstitials, self-interstitials and vacancies were produced in the ZrN and TaN layers. He atoms tended to combine with vacancies and grew into He bubbles (see Fig. 5(a)), resulting in amorphization in local regions of both nitride layers.

However, a strong defect-interface interaction can cause the migration of the point defects when the heterophase interfaces are introduced in the ZrN/TaN multilayered nanofilms. As revealed by computer simulation, the defect-interface interaction in the ZrN and TaN layers was not symmetric due to the difference in the vacancy formation energies in different nitrides. The vacancies in the ZrN layer would be preferentially absorbed by the heterophase interface^9^,^19^, due to the higher vacancy formation energy in the ZrN layer. Meanwhile, He atoms in the ZrN layer can migrate with the vacancies into the interface and grow into larger He bubbles because of their high binding energy with vacancies^7^,^9^. Therefore, a void-denude zone with very low density of the vacancies and He atoms results in a better crystallinity of the ZrN layers near the heterophase interfaces (see Fig. 5(b)).

The point defect clusters become thermodynamically unstable and decomposes into isolated defects when they grow in the interfaces^39^. The decomposition of the clusters may elevate the concentration of supersaturated interfacial point defects and increase the likelihood of their re-emission in the adjacent crystalline materials^7^. In the ZrN/TaN interface, the supersaturated vacancies and He atoms may be emitted back into the interior of the grains. However, the emission of the point defects (vacancies and He atoms) into both sides of the ZrN/TaN interface is not symmetric. The interfacial vacancies tend to be emitted into the TaN layer instead of the ZrN layers due to their lower vacancy formation energies in the TaN layer than that in the ZrN layer. Therefore, even if the likelihood of the He atom emission into each side of the interface is assumed to be equal, the emitted He atoms will move back because of the lack of sufficient vacancies on the ZrN side. The He atoms will then diffuse into the TaN layer driven by the high binding energy of He-vacancy (see Fig. 5(c)). Meanwhile, a large number of He bubbles nucleate with the increasing of the density of He atoms and vacancies, which will eventually result in a complete amorphization of the TaN layers (see Fig. 5(d)).

It is important to note that the migration mechanism of point defects proposed above seems to be contrary to the common sense that vacancies are considered to be unmovable at low temperatures^6^. Since the films were deposited without heating and stored at room temperature in this work, the elevated temperature (373 K–423 K) of the substrates during the films deposition can offer some energy to the vacancies diffusion. Furthermore, the grain size of the two nitrides in the multilayered nanofilms has decreased to about 5–10 nm (see Fig. 3(a2)), resulting in a substantial decrease in the distance between the vacancies and the grain boundaries. As He atoms were implanted into the multilayered nanofilms, the interstitial atoms would initially be captured by the grain boundaries. The interstitial-loaded grain boundaries can reduce the vacancy diffusion barrier and extend interaction range of vacancies and grain boundaries substantially, thus facilitating the migration of vacancies to the grain boundaries even at room temperature^6^,^18^. Because the grain boundaries have long been known as effective diffusion channels of the point defects, the vacancies can diffuse rapidly along the grain boundaries to the ZrN/TaN interfaces, and then into the TaN layers driven by the asymmetric defect-interface interaction. In fact, in addition to the grain boundaries, the large amount of the dislocations (see Fig. 2(a2) and (b2)) induced by He implantation also play a similar role in the diffusion of the vacancies. Therefore, it is reasonable to believe that the diffusion of the vacancies observed in this study is feasible.

Although the asymmetric defect-interface interaction in different phases have been found in several radiated nanocomposites^16^,^27^, its impact on the microstructural stability in radiation environment seems to be underestimated. For example, when designing radiation tolerant materials, a small distance between the heterophase interfaces is usually expected to maximize the sink effect of the interfaces on the radiation-induced point defects^9^,^28^. However, the supersaturated point defects would accumulate on the heterophase interfaces with the increasing of the radiation flux. In this extreme case, it was unknown whether the sink efficiency of the heterophase interfaces would be exhausted. It was also unclear about the microstructure response to the interaction of the point defects and the heterophase interface. Findings of this study show that the point defects can migrate from one phase to the other phase assisted by the asymmetric defect-interface interaction, resulting in a better crystallinity in one phase and a severe amorphization in the other phase.

Similar phenomena were also observed in ODS steels. The preferential amorphization of the oxides dispersed in ODS steel was often triggered by ion radiation because the oxides acted as effective sinks for trapping point defects^40^,^41^,^42^. Therefore, it is reasonable to believe that the asymmetric interaction of point defects and heterophase interfaces has a significant impact on the microstructure evolution of the radiation tolerant materials.

In summary, He atoms were implanted into the ZrN/TaN multilayered nanofilms to investigate the interaction between the point defects and the heterophase interfaces. The experimental observation and computer simulation show that the point defect-interface interaction on the two sides of the heterophase interfaces is not symmetric due to the variation of vacancy formation energies in different phases. This asymmetric defect-interface interaction caused the migration of He bubbles from the ZrN layers to the TaN layers, resulting in a complete amorphization of the TaN layers in the ZrN/TaN multilayered nanofilms. These findings suggest that the asymmetric defect-interface interaction can produce unexpected microstructure changes in materials during radiation. To design a stable radiation tolerant material, the differences in the vacancy formation energies of different phases of the material should be carefully considered.

## Methods

### Samples preparation

The ZrN(30 nm)/TaN(30 nm) multilayered nanofilms, ZrN and TaN monofilms with He implantation were deposited on single crystal Si (100) substrates by a radio frequency (RF) reactive magnetron sputtering system, respectively. Helium atoms were implanted into the films with little incident energy during depositing. This is different from the commonly used He implantation in the form of ion radiations. Because the radiation is not an equilibrium process, it can complicate the execution of experiments and the interpretation of the results due to the fact that the time, dose and depth dependence of radiation damage is complex^6^. High-flux of He atoms (5 × 10^17^–1.0 × 10^18^ atoms·cm^−2^) can be introduced uniformly into the whole films by the physical vapor deposition method, simplifying the observation and the interpretation of the experimental results^43^,^44^.

The total thickness of all the He-implanted films was kept at about 1 μm. A pure zirconium plate (99.995%) and a pure tantalum plate (99.995%) were used as targets. The target size was 65 mm in diameter, and the distance between the target and the substrate was maintained at 50 mm. The deposition of films was conducted in a stainless-steel vacuum chamber which was evacuated by a turbomolecular pump to 1.0 × 10^−4^ Pa prior to deposition. The substrates were cleaned ultrasonically with alcohol before being placed into the vacuum chamber, and then cleaned again by applying RF power of 200 W under Ar (purity of 99.999%) for 10 min prior to film deposition. The substrate holder was neither cooled nor heated during sputtering. The substrate temperature, which was monitored by a thermocouple placed on the substrate holder, varied from 373 K to 423 K during the deposition of the films. The target area was pre-sputtered for 10 min to remove possible impurities from the surface.

During the deposition of the He-implanted films, pure Ar, N_2_ and He were introduced into the chamber with influx rates of 45 sccm (standard cubic centimeter per minute), 25 sccm and 30 sccm, respectively. The working pressure during deposition was maintained at 0.8 Pa. With a similar procedure, multilayered ZrN (30 nm)/TaN (30 nm) nanofilms, ZrN and TaN monofilms without He (about 1 μm in thickness) were deposited on the Si substrates as control samples. During the deposition of the films without He implantation, pure Ar and N_2_ were introduced into the chamber with an influx rate of 42 sccm and 25 sccm, respectively, and the working pressure was kept at 0.3 Pa.

### Microstructure characterization

X-ray diffraction measurements were performed to ascertain the crystallographic structure of the nanofilms using a RigakuD/Max-3A X-ray diffractometer with the Cu-Kα radiation source operated at 40 kV and 40 mA. A high-resolution transmission electron microscopy (HRTEM, Tecnai F30, FEI) was employed to analyze the microstructures and interfaces characteristics of the nanofilms.

### Computational details

The interactions between the vacancy and the heterophase interface in ZrN/TaN systems were studied by using the first-principles calculations. The vacancy formation energies at different sites near the ZrN(111)/TaN(111) interface were calculated within the density functional theory framework as implemented in VASP code^45^,^46^,^47^. The projector augmented–wave (PAW) method to describe the ion-electron interaction^48^, and the Perdew-Burke-Ernzerhof (PBE) version of generalized gradient approximation (GGA) function were applied^49^. The energy cutoff of 400 eV was used for the plane-wave base set expansion. The convergence criteria for total energy and residual force during geometry relaxation were 10^−5^ eV per cell and 0.02 eV/Å for each atom, respectively.

The preferential orientation was determined to be [111] direction of the face-centered cubic (NaCl type, space group Fm-3m) geometry of TaN and ZrN films based on the experimental observations (Figs 1 and 2(a2)). The lattice parameter *a* was found to be 4.42 and 4.59 Å for TaN and ZrN films, respectively. An orthogonal supercell with the original [111] direction along *z* axis were built, with the new lattice parameter equal to 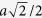, 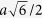, 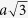 along *x, y*, and *z* axis, respectively. The interface model was constructed by putting two repetitions of each new [111] oriented supercell of both ZrN and TaN films together. The constructed supercell was fully relaxed before creating a vacancy. The resulted *a* was 4.52 Å, close to the value of ZrN. In order to consider the presence of vacancies, the periodicity of p(5 × 3) in the plane parallel to the new interface model was used. This process resulted in a very large supercell containing 720 atoms before a vacancy was created, as shown in Fig. 4(a). The Γ-point only and the k-points grid of 3 × 3 × 1 used for the Brillouin zone integration were sampled with Monkhorst-Pack method for geometry relaxation and total energy calculations, respectively^50^. The nitrogen vacancy formation energies E_vac_ were calculated according to


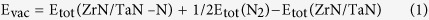


The E_tot_(ZrN/TaN-N), E_tot_(N_2_), –and E_tot_(ZrN/TaN) are the total energies of the supercell interface model after removing one nitrogen atom at some certain site, of the isolated nitrogen gas molecule, and of the pristine ZaN/TaN interface supercell model, respectively. All the geometries were relaxed but with the supercell lattice fixed.

## Additional Information

**How to cite this article:** Lao, Y. *et al*. Asymmetric interaction of point defects and heterophase interfaces in ZrN/TaN multilayered nanofilms. *Sci. Rep.* **7**, 40044; doi: 10.1038/srep40044 (2017).

**Publisher's note:** Springer Nature remains neutral with regard to jurisdictional claims in published maps and institutional affiliations.
